# Hypoxia increases the metastatic ability of breast cancer cells via upregulation of CXCR4

**DOI:** 10.1186/1471-2407-10-225

**Published:** 2010-05-21

**Authors:** Patricia A Cronin, Jiang H Wang, H Paul Redmond

**Affiliations:** 1 Department of Academic Surgery, University College Cork, Cork University Hospital, Cork, Ireland

## Abstract

**Background:**

Chemokine SDF1α and its unique receptor CXCR4 have been implicated in organ-specific metastases of many cancers including breast cancer. Hypoxia is a common feature of solid tumors and is associated with their malignant phenotype. We hypothesized that hypoxia would upregulate CXCR4 expression and lead to increased chemotactic responsiveness to its specific ligand SDF1α.

**Methods:**

Three breast cancer cell lines MDA-MB-231, MCF7 and 4T1 were subjected to 48 hrs of hypoxia or normoxia. Cell surface receptor expression was evaluated using flow cytometry. An extracellular matrix invasion assay and microporous migration assay was used to assess chemotactic response and metastatic ability.

**Results:**

CXCR4 surface expression was significantly increased in the two human breast cancer cell lines, MDA-MB-231 and MCF7, following exposure to hypoxia. This upregulation of CXCR4 cell surface expression corresponded to a significant increase in migration and invasion in response to SDF1-α *in vitro*. The increase in metastatic potential of both the normoxic and the hypoxic treated breast cancer cell lines was attenuated by neutralization of CXCR4 with a CXCR4 neutralizing mAb, MAB172 or a CXCR4 antagonist, AMD3100, showing the relationship between CXCR4 overexpression and increased chemotactic responsiveness.

**Conclusions:**

CXCR4 expression can be modulated by the tissue microenvironment such as hypoxia. Upregulation of CXCR4 is associated with increased migratory and invasive potential and this effect can be abrogated by CXCR4 inhibition. Chemokine receptor CXCR4 is a potential therapeutic target in the adjuvant treatment of breast cancer.

## Background

Breast cancer is the most common malignancy in women, characterized by a distinct pattern of metastasis involving regional lymph nodes, bone marrow, lung and liver. About 1 million cases of breast cancer are detected each year in the world [1]. Although early stage breast cancers are not life threatening, development of metastatic breast cancer is responsible for the majority of cancer-related death. Metastasis is the result of several sequential steps and represents a highly organized, non-random and organ-selective process [2]. A wide number of molecules such as cytokines, chemokines and their receptors, and growth factors have been implicated to be responsible for the metastatic property of breast cancer cells [3-9]. However, the precise cellular and molecular mechanisms that determine primary tumour growth and the directional migration and invasion of tumour cells into the secondary organs have yet to be established.

Hypoxia is the result of an imbalance between oxygen delivery and oxygen consumption resulting in the reduction of oxygen tension below the normal level for a specific tissue [10]. Oxygen tensions have been measured in several cancer types, using eppendorf histography electrodes showing a range of values between 0 and 20 mmHg in tumour tissues, which were significantly lower than those of the adjacent tissue (24 - 66 mmHg) [11-13]. In breast cancers of stages T1b-T4, measurement of oxygen tension revealed a median pO2 of 28 mmHg compared with 65 mmHg in normal breast tissue [14]. Hypoxia in solid tumours like breast cancer is felt to be due to the tumour outgrowing the existing vasculature. Under these hypoxic conditions, numerous cellular mechanisms are compromised and an adaptive response occurs which allows cancer cells to adapt to this hostile environment. This renders them more resistant and with improved ability to survive and even proliferate, promoting tumour development [15].

Hypoxia-inducible factor (HIF) is a transcription factor that responds to changes in available oxygen in the cellular environment [16]. HIF consists of two subunits, a α subunit whose level increases during hypoxia and a β subunit that is constitutively expressed [17]. Although HIF-1α expression may also be influenced by other pathways, a significant correlation between oxygen tension and HIF-1α has been reported in cervical cancer, suggesting that HIF-1α might be used as a surrogate for tumour hypoxia [18]. By using HIF-1α as a marker for hypoxia, approximately 25-40% of all invasive breast cancer samples are hypoxic; the frequency of HIF-1α-positive cells increases in parallel with increasing pathologic stage and is associated with a poor prognosis [19-21].

Clear-cell renal cell carcinoma (ccRCC), the most frequent subtype of renal cancer, is characterized by inactivation of the von Hippel-Lindau (VHL) tumour suppressor gene in about 70% of the tumours. The VHL protein binds to HIF and targets it for ubiquitination and degradation. Therefore, loss of VHL in these tumours leads to persistently elevated levels of HIF expression [22]. Loss of VHL function in ccRCC also results in strongly enhanced transcription of the HIF-inducible, G-protein-coupled, CXC motif, chemokine receptor 4 (CXCR4), and its cognate, stromal derived factor1α (SDF1α) [22,23].

CXCR4 is a G-protein coupled receptor that is expressed constitutively in a wide variety of normal tissues, including lymphatic tissues, thymus, brain, spleen, stomach, and small intestine [24]. This receptor is also expressed in normal stem cells from a variety of tissues, including mammary stem cells [25]. The fact that CXCR4 is present in normal mammary stem cells suggests that this molecule may be essential for stem cells that appear to be progenitors of breast carcinoma [26]. Signalling through CXCR4 activates a number of downstream effector molecules, including molecules that regulate key processes such as cell cycle control and apoptosis. The chemokine SDF1α is also expressed constitutively in a variety of tissues, including lung, liver, lymph nodes, bone marrow, and adrenal glands [24,27,28]. Of particular relevance to breast cancer, many of the organs with highest expression levels of SDF1α correlate with common sites of metastatic breast cancer, such as bone, liver lung and lymph nodes. Muller et al [9] investigated functions of chemokines and chemokine receptors in breast cancer. These investigators found that high levels of SDF1α are produced in many organs and tissues commonly affected by metastatic breast cancer, while CXCR4 appears to be expressed in human breast cancer cells and metastatic lesions. CXCR4 signalling in response to SDF1α induces chemotaxis and migration of breast cancer cells. In a mouse model of breast cancer, neutralizing antibodies to CXCR4 significantly limited metastases to lymph nodes and lung [9]. Their data were the first to identify a key function for SDF1α-CXCR4 in metastatic breast cancer. Our hypothesis was that the hypoxia commonly found in primary breast cancers would induce an increased expression of CXCR4, and that this increase in CXCR4 expression would increase breast cancer cells metastatic ability.

## Methods

### Cell lines and cultures

MCF7 and MDA-MB-231, two human breast cancer cell lines, and 4T1, a murine breast cancer cell line, were maintained in Dulbecco-modified Eagle medium (DMEM), supplemented with 10% fetal calf serum, penicillin (100 units/ml), streptomycin (100 μg/ml), and glutamine (2 mM). Cells were grown at 37°C in a humidified atmosphere with 5% CO_2_. The cells in hypoxic experiments were exposed to 2% O_2 _with 5% CO_2 _at 37°C for 48 hrs. All culture medium and reagents for cell cultures were purchased from Invitrogen Life Technologies (Paisley, Scotland, UK).

### Cell surface expression of CXCR4

After being cultured in either normoxic condition or hypoxic condition for 48 hrs, cells were stained with PE-conjugated anti-CXCR4 mAb (FAB170P, Clone 12G5) (R&D Systems, Minneapolis, MN). PE-conjugated mouse IgG_2a _isotype-matched mAb (IC003P, R&D Systems) was used as the negative control. FACScan analysis was performed from at least 5,000 events for detecting cell surface expression of CXCR4 using CellQuest software (BD Biosciences, San Jose, CA).

### Tumour cell migration and invasion assay

Tumour cell migration through a microporous membrane and invasion through an extracellular matrix were assessed based on the Boyden chamber principle using the fluorescent CyQuant GR dye (Chemicon, Temecula, CA). Cells were incubated with culture medium as the control, 10 μg/ml control IgG_2b _mAb (MAB004, R&D Systems), 10 μg/ml CXCR4 blocking mAb (MAB172, R&D Systems), or 100 nM CXCR4 antagonist AMD3100 (Sigma-Aldrich, St. Louise, MO) for 1 hr, and then plated onto the top chamber. Culture medium containing 100 ng/ml of either recombinant mouse SDF1α (460-SD, R&D Systems) or recombinant human SDF1α (350-NS, R&D Systems) was added into the lower chamber. The plate was incubated at 37°C, 5% CO_2 _for 18 hrs. The migrated or invaded cells were dislodged from the underside of the chamber using a detachment solution. A lysis buffer and dye solution was added and the plate was read with a fluorescence plate reader using a 480/520 nm filter set (Thermo Labsystems, Cheshire, England, UK).

### Cell viability

Cell viability was assessed by measuring the ability of cells to metabolize MTT (Sigma-Aldrich), a water-soluble tetrazolium salt, into a water insoluble formazan product. In brief, tumour cells (5 × 10^3^/well) were plated into 96-well plates at 37°C, 5% CO_2 _for 12 hrs. Cells were then treated with culture medium, 10 μg/ml control IgG_2b _mAb (MAB004, R&D Systems), 10 μg/ml CXCR4 blocking mAb (MAB172, R&D Systems), or 100 nM AMD3100 (Sigma-Aldrich) at varying doses. After 24 hrs, 50 μl of 1 mg/ml of MTT solution was added to each well and incubated for 3 hrs. The supernatant was then gently removed and 100 μl DMSO (Sigma-Aldrich) added to each well. The OD value in each well was measured with a micro-plate reader (Dynatech Laboratories, Chantilly, VA) at absorption wavelength of 570 nm with a reference wavelength of 650 nm.

### Statistical analysis

All data are presented as the mean ± SD. The significance of the difference was analysed with a two-sided Student's *t *test, and a p value < 0.05 was considered statistically significant.

## Results

### Hypoxia facilitates breast cancer cell migration and invasion

**Figure 1:**
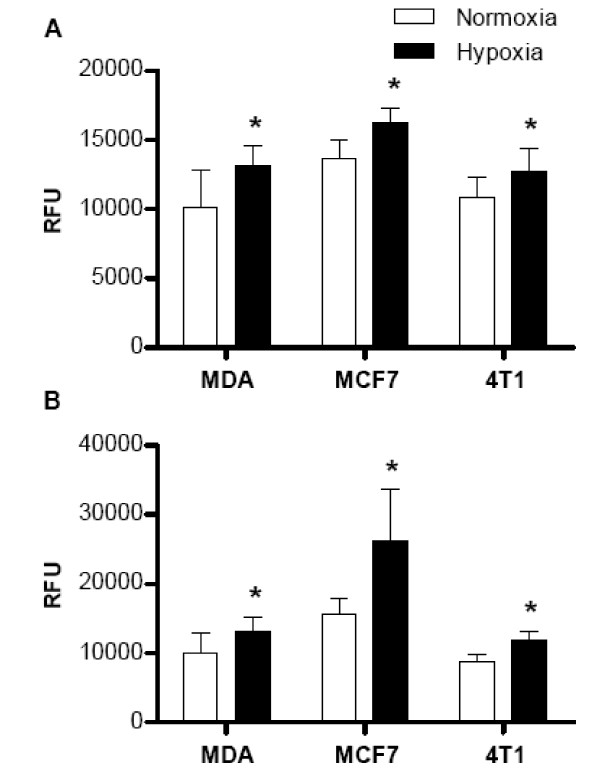
**Migration of breast cancer cells through a microporus membrane (A) and invasion through an extracellular matrix (B) in response to SDF1α in normoxia and hypoxia conditions**. Three cell lines MDA-MB-231, MCF7, and 4T1 cells were exposed to either normoxia or hypoxia (2% O_2 _with 5% CO_2_) for 48 hrs. Cell migration and invasion were assessed as described in the Methods and expressed as relative fluorescence units (RFU). Data are presented as the mean ± SD of duplicate samples and are representative of three independent experiments. *p < 0.05 versus normoxia-treated cells.

To examine the effect of hypoxia on breast cancer cell metastatic ability, two human breast cancer cell lines MCF7 and MDA-MB-231, and one murine breast cancer cell line 4T1 were exposed to either normoxia or hypoxia for 48 hrs. Breast cancer cell migration through a microporous membrane and invasion through an extracellular matrix were assessed under both normoxic and hypoxic conditions. It was observed that exposure of these three breast cancer cell lines to hypoxic conditions resulted in significantly increased migration (Figure 1A) and invasion (Figure 1B) in vitro.

**Figure 2:**
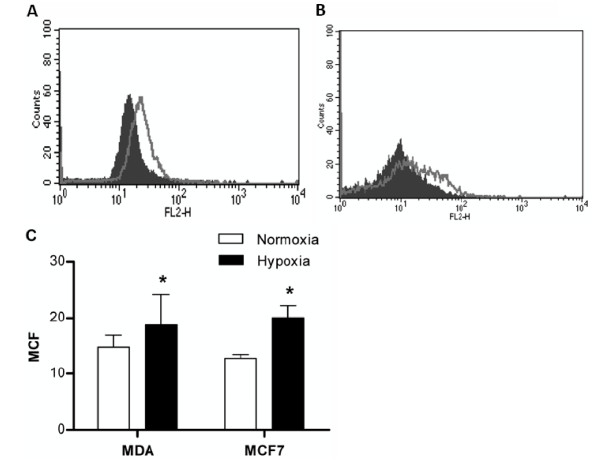
**Cell surface expression of CXCR4 in normoxia and hypoxia treated MDA-MB-231 (A) and MCF7 (B) cells**. Human breast cancer cell lines, MDA-MB-231 and MCF7 cells were exposed to either normoxia or hypoxia (2% O_2 _with 5% CO_2_) for 48 hrs. Cell surface expression of CXCR4 on breast cancer cells was assessed by FACScan analysis, and expressed as mean channel fluorescence (MCF). (A-B) Results shown represent one experiment from a total of three independent experiments. The grey filled indicates CXCR4 expression on normoxia-treated cells, whereas the grey line indicates CXCR4 expression on hypoxia-treated cells. (C) Data are expressed as the mean ± SD of three separate experiments. *p < 0.05 versus normoxia-treated cells.

Following confirmation of increased metastatic ability in hypoxia-treated breast cancer cells, we wished to investigate whether CXCR4 played a role in this increased capacity, as CXCR4 has been implicated in breast cancer metastases and linked to tumours with increased levels of HIF1α (a surrogate of hypoxia). We again treated cells to the same hypoxic and normoxic conditions and assessed cell surface expression of CXCR4 by FACScan analysis. Exposure of two human breast cancer cell lines MDA-MB 231 and MCF7 to hypoxia led to a statistically significant increase in cell surface expression of CXCR4 by 27.5% (p < 0.05) and 67.5% (p < 0.05), respectively, when compared to cells exposed to normoxia (Figure 2A-C).

### Blocking CXCR4 attenuates breast cancer cell migration and invasion

**Figure 3:**
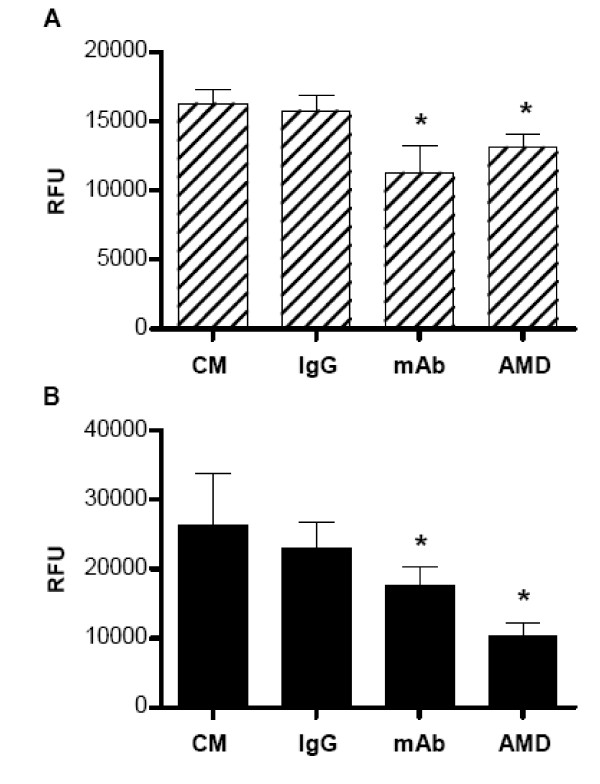
**The effect of CXCR4 inhibition on cell migration (A) and invasion (B) in hypoxia-treated breast cancer cells**. Human breast cancer cell line, MCF7 cells were exposed to hypoxia (2% O_2 _with 5% CO_2_) for 48 hrs, and further incubated with either a CXCR4 blocking mAb, MAB172 (mAB, 10 μg/ml), or a CXCR4 antagonist, AMD3100 (AMD, 100 nM) for 1 hr. Culture medium (CM) and control IgG_2b _mAb, MAB004 (IgG, 10 μg/ml) were used as the control. Cell migration and invasion were assessed as described in the Methods and expressed as (relative fluorescence units RFU). Data are presented as the mean ± SD of duplicate samples and are representative of three independent experiments. *p < 0.05 versus CM or IgG treated cells.

To determine if the increased metastatic potential seen following hypoxia treatment was associated with the increased cell surface expression of CXCR4, we repeated the migration and invasion experiments in two human breast cancer cells lines, MDA-MB 231 and MCF7. Cells were exposed to either 48 hrs of the same normoxic or hypoxic conditions. Before assessment of cell migration and invasion, both MDA-MB-231 and MCF7 cells were incubated with either a CXCR4 blocking mAb, MAB172 (10 μg/ml), or a CXCR4 antagonist, AMD3100 (100 nM) for 1 hr. Culture medium and control IgG_2b _mAb, MAB004 (10 μg/ml) were used as the control. It was observed that attenuation of CXCR4 with either the blocking mAb, MAB172 or the antagonist, AMD3100 led to statistically significant attenuation of breast cancer cell migration in response to SDF1α in MCF7 (Figure 3A) and MDA-MB-231 (data not shown) cells. This effect was not seen with the isotype control mAb.

Similarly, CXCR4 inhibition with the CXCR4 blocking mAb led to statistically significant attenuation of cell invasion through an extracellular matrix in response to SDF1α observed in hypoxia-treated MCF7 (Figure 3B) and MDA-MB-231 (data not shown) cells. The CXCR4 antagonist, AMD3100 was also observed to have a similar effect as the CXCR4 blocking mAb with attenuation of extracellular matrix invasion of hypoxia-treated MCF7 (Figure 3B) and MDA-MB-231 (data not shown) cells.

**Figure 4:**
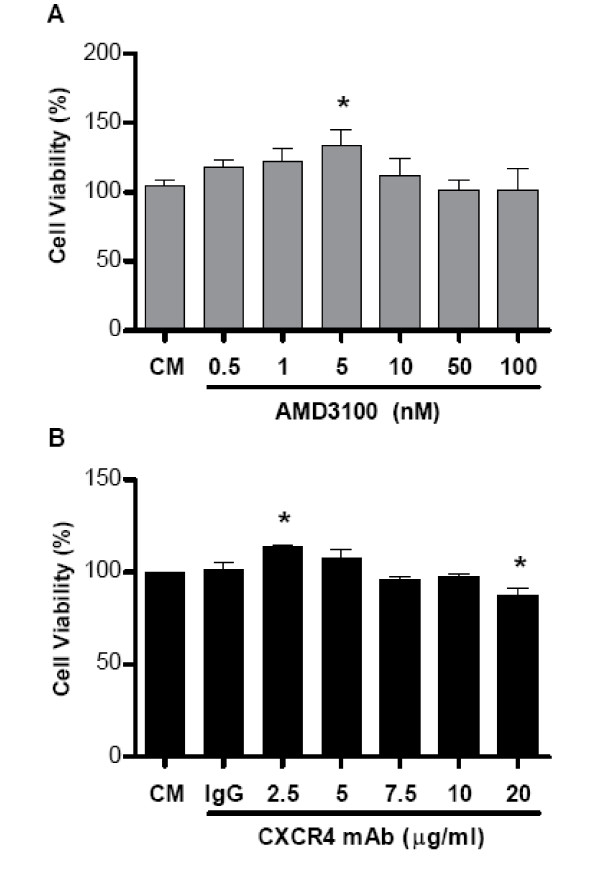
**Cell viability of MCF7 breast cancer cells following treatment with either AMD3100 or the CXCR4 blocking mAb**. Cells were treated with (A) CXCR4 antagonist AMD3100 (0.5 - 100 nM) and (B) the blocking mAb, MAB172 (2.5 - 20 μg/ml) for 24 hrs at 5% CO_2 _at 37°C. Control IgG_2b _mAb, MAB004 (10 μg/ml) was used as the control. Data are expressed as the mean ± SD of duplicate samples and are representative of three independent experiments. *p < 0.05 versus (A) culture medium (CM) or (B) control mAb (IgG) treated cells.

To assure that the effects of CXCR4 attenuation on breast cancer cell migration and invasion was not due to decreased cell viability resulted from the treatment with the CXCR4 blocking mAb or the CXCR4 antagonist; we assessed the effect of CXCR4 blockade on cell viability with an MTT assay. AMD3100 was tested at varying concentrations from 0.5 to 100 nM and the blocking mAb was tested at concentrations from 2.5 to 20 μg/ml. As shown in Figure 4A and 4B, treatment of breast cancer cells with AMD3100, the blocking mAb or the isotype control mAb did not significantly affect cell viability *in vitro*. Lower doses of CXCR4 blockade seemed to have a mild proliferative effect and this was significant at a dose of 5 nM of AMD and at a dose of 2.5 μg/ml of the blocking mAb. The higher dose of 20 μg/ml of the mAb did show a significant negative effect on cell viability, but the treatment dose of 10 μg/ml did not.

## Discussion

It has been proposed that molecules regulating the metastatic dissemination of tumour cells in an organ specific manner would need to fulfil certain criteria [2,29]. They would have to be constitutively expressed in the target organs of metastasis. Adhesion of the circulating tumour cells to the endothelium and their transendothelial migration would need to be promoted. These molecules would need to be capable of mediating the invasion of tumour cells into tissues that would provide supportive microenvironments. Lastly, this process would require the expression of a distinct receptor repertoire by the target cells, depending on their metastatic profile. Given their well-established roles in leukocyte trafficking and homeostasis, chemokines are perfectly positioned to fulfil these criteria [30-37].

CXCR4 with its unique ligand SDF1α has been and continues to be a source of investigation into the organ specific metastases of several types of cancer including breast cancer. Muller et al [9] were the first to investigate the functions of chemokines and chemokine receptors in breast cancer. They found that high levels of SDF1α are produced in many organs and tissues commonly affected by metastatic breast cancer, while CXCR4 appears to be expressed in human breast cancer cells and metastatic lesions. CXCR4 signalling in response to SDF1α was found to induce chemotaxis and migration of breast cancer cells. These data were the first to elucidate key roles for the CXCR4-SDF1α receptor-ligand axis in metastatic breast cancer. The hypothesis now proposed holds that organ-specific attractant molecules such as SDF1α stimulate the migrating tumour cells to invade the walls in blood vessels and enter the target organs. This theory is widely known as "chemoattraction" theory which is similar with Paget's original paper looking at the site specific pattern of breast cancer metastases proposing "seed and soil theory" [38].

Hypoxia is a primary factor in the pathology of many disease states, including solid tumours [12-14]. In this paper we propose that in solid tumours such as breast cancer, in addition to genetic alterations such as mutations of VHL [22], PTEN [39], or p53 [40] that are associated with increased levels of HIF1 transcriptional activity, tumour microenvironmental hypoxia may increase CXCR4 expression and thus the metastatic potential of cancer cells. The results in this paper support other findings in the literature that suggest that hypoxia plays a key role in CXCR4 expression. Schioppa T et al [41] also looked at the role of oxygen levels on CXCR4 expression. They found that low oxygen concentration induced high expression of SDF1α and CXCR4, in different cell types (monocytes, monocyte-derived macrophages, tumour-associated macrophages, endothelial cells, and cancer cells). Our data support these findings showing that breast cancer cells are sensitive to the microenvironment, in particular oxygen levels which can lead to upregulation of CXCR4 cell surface expression.

We further demonstrated that upregulation of CXCR4 in breast cancer cells secondary to hypoxia is associated with increased chemotactic ability and metastatic potential, and that this effect can be abrogated by CXCR4 attenuation. This finding may indicate a potential therapeutic role for CXCR4 inhibition. Certainly, in vivo studies [9,42] have confirmed this theory with CXCR4 inhibition in mouse models of cancer showing decreased metastases. Immunhistochemistry studies of primary breast tumours have also demonstrated a relationship between CXCR4 expression and metastases [43-47]. Tumours with high expression of CXCR4 are associated with more aggressive phenotypes showing increased rates of distant metastases and poorer clinical outcome. Organ-specific metastases to liver [46] and bone marrow [47] have been shown again consistent with the "chemoattraction" theory. Further reason to believe that interference with the CXCR4-SDF1α receptor-ligand axis could be of potential therapeutic benefit in the treatment or prevention of breast cancer metastases.

The perioperative period may afford a window of opportunity for treatment using interference of the CXCR4-SDF1α axis. There are many factors during the perioperative period that are thought to affect tumour development [48]. One of these is the possibility of increased circulating tumour cells postoperatively [49,50]. Therefore, we would propose that treatment with CXCR4 antagonists such as AMD3100 in the perioperative period would prevent the migration and invasion of CXCR4 expressing breast cancer cells into organs of metastases that are rich in SDF1α. AMD3100 is safe, in December 2008, Plerixafor, the new trade name for AMD3100, was approved by the FDA of United States for use in patients with non-Hodgkin's lymphoma and multiple myeloma [51]. It is used in combination with granulocyte colony-stimulating factor in these patients to mobilize haematopoietic stem cells to the peripheral blood for collection and subsequent autologous transplantation.

## Conclusion

In summary, CXCR4-SDF1α receptor-ligand axis plays an important role in the metastatic ability of breast cancer cells. CXCR4 is expressed on breast cancer cells and exposure to hypoxia upregulated this expression. From the literature we know that hypoxic breast cancer primary tumours correlate with later stage tumours and metastases and are associated with patients of poor prognosis. We propose that one of the mechanisms underlying this increased metastatic ability and poorer prognosis is that tumour hypoxia through upregulation of CXCR4 cell surface expression leads to increased metastatic potential of breast cancer cells. We would also suggest that further work in this area should look at utilising the perioperative window as the possible timing of interference in this axis as a potential metastases preventative strategy.

## Competing interests

The authors declare that they have no competing interests.

## Contributing Authors

PAC and JHW carried out the experiments described in the study while the study was designed and analysed by PAC, JHW and HPR. All authors read and approved the final manuscript.

## Pre-publication history

The pre-publication history for this paper can be accessed here:

http://www.biomedcentral.com/1471-2407/10/225/prepub

## References

[B1] McPherson K Steel CM Dixon JM ABC of breast diseases. Breast cancer-epidemiology, risk factors, and genetics BMJ 2000 321 624 8 10.1136/bmj.321.7261.624 10977847 PMC1118507

[B2] Nicolson GL Paracrine and autocrine growth mechanisms in tumor metastasis to specific sites with particular emphasis on brain and lung metastasis Cancer Metastasis Rev 1993 12 325 43 10.1007/BF00665961 8281616

[B3] Youngs SJ Ali SA Taub DD Rees RC Chemokines induce migrational responses in human breast carcinoma cell lines Int J Cancer 1997 71 257 66 10.1002/(SICI)1097-0215(19970410)71:2<257::AID-IJC22>3.0.CO;2-D 9139852

[B4] Verbeek BS Adriaansen-Slot SS Vroom TM Beckers T Rijksen G Overexpression of EGFR and c-erbB2 causes enhanced cell migration in human breast cancer cells and NIH3T3 fibroblasts FEBS Lett 1998 425 145 50 10.1016/S0014-5793(98)00224-5 9541025

[B5] Kim H Muller WJ The role of the epidermal growth factor receptor family in mammary tumorigenesis and metastasis Exp Cell Res 1999 253 78 87 10.1006/excr.1999.4706 10579913

[B6] Hilakivi-Clarke L Estrogens, BRCA1, and breast cancer Cancer Res 2000 60 4993 5001 11016617

[B7] Hyder SM Chiappetta C Stancel GM Pharmacological and endogenous progestins induce vascular endothelial growth factor expression in human breast cancer cells Int J Cancer 2001 92 469 73 10.1002/ijc.1236 11304678

[B8] McEarchern JA Kobie JJ Mack V Wu RS Meade-Tollin L Arteaga CL Dumont N Besselsen D Seftor E Hendrix MJ Katsanis E Akporiaye ET Invasion and metastasis of a mammary tumor involves TGF-beta signaling Int J Cancer 2001 91 76 82 10.1002/1097-0215(20010101)91:1<76::AID-IJC1012>3.0.CO;2-8 11149423

[B9] Müller A Homey B Soto H Ge N Catron D Buchanan ME McClanahan T Murphy E Yuan W Wagner SN Barrera JL Mohar A Verástegui E Zlotnik A Involvement of chemokine receptors in breast cancer metastasis Nature 2001 410 50 6 10.1038/35065016 11242036

[B10] Lundgren K Holm C Landberg G Hypoxia and breast cancer: prognostic and therapeutic implications Cell Mol Life Sci 2007 64 3233 47 10.1007/s00018-007-7390-6 17957335 PMC11136324

[B11] Brizel DM Rosner GL Prosnitz LR Dewhirst MW Patterns and variability of tumor oxygenation in human soft tissue sarcomas, cervical carcinomas, and lymph node metastases Int J Radiat Oncol Biol Phys 1995 32 1121 5 7607933 10.1016/0360-3016(95)00106-9

[B12] Vaupel P Hockel M Mayer A Detection and characterization of tumor hypoxia using pO2 histography Antioxid Redox Signal 2007 9 1221 35 10.1089/ars.2007.1628 17536958

[B13] Vaupel P Okunieff P Neuringer LJ Blood flow, tissue oxygenation, pH distribution, and energy metabolism of murine mammary adenocarcinomas during growth Adv Exp Med Biol 1989 248 835 45 2782192 10.1007/978-1-4684-5643-1_95

[B14] Vaupel P Schlenger K Knoop C Hockel M Oxygenation of human tumors: evaluation of tissue oxygen distribution in breast cancers by computerized O2 tension measurements Cancer Res 1991 51 3316 22 2040005

[B15] Harris AL Hypoxia - a key regulatory factor in tumour growth Nat Rev Cancer 2002 2 38 47 10.1038/nrc704 11902584

[B16] Smith TG Robbins PA Ratcliffe PJ The human side of hypoxia-inducible factor Br J Haematol 2008 141 325 34 18410568 10.1111/j.1365-2141.2008.07029.x PMC2408651

[B17] Semenza GL Hypoxia-inducible factor 1: oxygen homeostasis and disease pathophysiology Trends Mol Med 2001 7 345 50 10.1016/S1471-4914(01)02090-1 11516994

[B18] Bos R Groep P van der Greijer AE Shvarts A Meijer S Pinedo HM Semenza GL van Diest PJ Wall E van der Levels of hypoxia-inducible factor-1α independently predict prognosis in patients with lymph node negative breast carcinoma Cancer 2003 97 1573 1581 10.1002/cncr.11246 12627523

[B19] Gruber G Greiner RH Hlushchuk R Aebersold DM Altermatt HJ Berclaz G Djonov V Hypoxia-inducible factor 1 alpha in high-risk breast cancer: An independent prognostic parameter? Breast Cancer Res 2004 6 R191 R198 10.1186/bcr775 15084243 PMC400672

[B20] Vleugel MM Greijer AE Shvarts A Groep P van der van Berkel M Aarbodem Y van Tinteren H Harris AL van Diest PJ Wall E van der Differential prognostic impact of hypoxia induced and diffuse HIF-1α expression in invasive breast cancer J Clin Pathol 2005 58 172 177 10.1136/jcp.2004.019885 15677538 PMC1770566

[B21] Trastour C Benizri E Ettore F Ramaioli A Chamorey E Pouysségur J Berra E HIF-1alpha and CA IX staining in invasive breast carcinomas: Prognosis and treatment outcome Int J Cancer 2007 120 1451 1458 10.1002/ijc.22436 17245699

[B22] Staller P Sulitkova J Lisztwan J Moch H Oakeley EJ Krek W Chemokine receptor CXCR4 down-regulated by von Hippel- Lindau tumour suppressor pVHL Nature 2003 425 307 311 10.1038/nature01874 13679920

[B23] Zagzag D Krishnamachary B Yee H Okuyama H Chiriboga L Ali MA Melamed J Semenza GL Stromal cell-derived factor-1α and CXCR4 expression in hemangioblastoma and clear cell-renal cell carcinoma: von Hippel-Lindau loss-of-function induces expression of a ligand and its receptor Cancer Res 2005 65 6178 6188 10.1158/0008-5472.CAN-04-4406 16024619

[B24] Chang L Karin M Mammalian MAP kinase signalling cascades Nature 2001 410 37 40 10.1038/35065000 11242034

[B25] Fresno Vara J Casado E de Castro J Cejas P Belda-Iniesta C Gonzalez-Baron M PI3K/Akt signalling pathway and cancer Cancer Treat Rev 2004 30 193 204 10.1016/j.ctrv.2003.07.007 15023437

[B26] Zhou Y Larsen P Hao C Yong V CXCR4 is a major chemokine receptor on glioma cells and mediates their survival J Biol Chem 2002 277 19481 19487 10.1074/jbc.M206222200 12388552

[B27] Vlahakis S Villasis-Keever A Gomez T Vanegas M Vlahakis N Paya C G protein-coupled chemokine receptors induce both survival and apoptotic signaling pathways J Immunol 2002 169 5546 5554 12421931 10.4049/jimmunol.169.10.5546

[B28] Vila-Coro A Rodriguez-Frade J Martin De Ana A Moreno-Ortiz M Martinez-A C Mellado M The chemokine SDF-1alpha triggers CXCR4 receptor dimerization and activates the JAK/STAT pathway Fed Am Soc Exp Biol J 1999 13 1699 1710 10506573

[B29] Wang JM Deng X Gong W Su S Chemokines and their role in tumor growth and metastasis J Immunol Methods 1998 220 1 17 10.1016/S0022-1759(98)00128-8 9839921

[B30] Zlotnik A Yoshie O Chemokines: a new classification system and their role in immunity Immunity 2000 12 121 127 10.1016/S1074-7613(00)80165-X 10714678

[B31] Campbell JJ Butcher EC Chemokines in tissue-specific and microenvironment-specific lymphocyte homing Curr Opin Immunol 2000 12 336 341 10.1016/S0952-7915(00)00096-0 10781407

[B32] Morales J Homey B Vicari AP Hudak S Oldham E Hedrick J Orozco R Copeland NG Jenkins NA McEvoy LM Zlotnik A CTACK, a skin-associated chemokine that preferentially attracts skin-homing memory T cells Proc Natl Acad Sci USA 1999 96 14470 14475 10.1073/pnas.96.25.14470 10588729 PMC24460

[B33] Homey B Wang W Soto H Buchanan ME Wiesenborn A Catron D Müller A McClanahan TK Dieu-Nosjean MC Orozco R Ruzicka T Lehmann P Oldham E Zlotnik A Cutting edge: the orphan chemokine receptor G protein-coupled receptor-2 (GPR-2, CCR10) binds the skin-associated chemokine CCL27 (CTACK/ALP/ILC) J Immunol 2000 164 3465 3470 10725697 10.4049/jimmunol.164.7.3465

[B34] Peled A Petit I Kollet O Magid M Ponomaryov T Byk T Nagler A Ben-Hur H Many A Shultz L Lider O Alon R Zipori D Lapidot T Dependence of human stem cell engraftment and repopulation of NOD/SCID mice on CXCR4 Science 1999 283 845 848 10.1126/science.283.5403.845 9933168

[B35] Förster R Schubel A Breitfeld D Kremmer E Renner-Müller I Wolf E Lipp M CCR7 coordinates the primary immune response by establishing functional microenvironments in secondary lymphoid organs Cell 1999 99 23 33 10.1016/S0092-8674(00)80059-8 10520991

[B36] Hedrick JA Zlotnik A Identification and characterization of a novel beta chemokine containing six conserved cysteines J Immunol 1997 159 1589 1593 9257816

[B37] Campbell JJ Hedrick J Zlotnik A Siani MA Thompson DA Butcher EC Chemokines and the arrest of lymphocytes rolling under flow conditions Science 1998 279 381 384 10.1126/science.279.5349.381 9430588

[B38] Paget S The distribution of secondary growths in cancer of the breast Lancet 1889 133 571 573 10.1016/S0140-6736(00)49915-0 2673568

[B39] Zundel W Schindler C Haas-Kogan D Koong A Kaper F Chen E Gottschalk AR Ryan HE Johnson RS Jefferson AB Stokoe D Giaccia AJ Loss of *PTEN *facilitates HIF-1-mediated gene expression Genes Dev 2000 14 391 396 10691731 PMC316386

[B40] Blagosklonny MV An WG Romanova LY Trepe J Fojo T Neckers L p53 inhibits hypoxia-inducible factor-stimulated transcription J Biol Chem 1998 273 11995 11998 10.1074/jbc.273.20.11995 9575138

[B41] Schioppa T Uranchimeg B Saccani A Biswas SK Doni A Rapisarda A Bernasconi S Saccani S Nebuloni M Vago L Mantovani A Melillo G Sica A Regulation of the chemokine receptor CXCR4 by hypoxia J Exp Med 2003 198 1391 1402 10.1084/jem.20030267 14597738 PMC2194248

[B42] Harvey JR Mellor P Eldaly H Lennard TW Kirby JA Ali S Inhibition of CXCR4-Mediated Breast Cancer Metastasis: A Potential Role for Heparinoids? Clin Cancer Res 2007 13 1562 70 10.1158/1078-0432.CCR-06-1987 17332302

[B43] Chu QD Panu L Holm N Li B Johnson L Zhang S High chemokine receptor CXCR4 level in triple negative breast cancer specimens predicts poor clinical outcome J Surg Res 2010 159 689 95 10.1016/j.jss.2008.09.020 19500800

[B44] Andre F Xia W Conforti R Wei Y Boulet T Tomasic G Spielmann M Zoubir M Berrada N Arriagada R Hortobagyi GN Hung MC Pusztai L Delaloge S Michiels S Cristofanilli M CXCR4 expression in early breast cancer and risk of distant recurrence Oncologist 2009 14 1182 8 10.1634/theoncologist.2009-0161 19939894

[B45] Holm NT Byrnes K Li BD Turnage RH Abreo F Mathis JM Chu QD Elevated levels of chemokine receptor CXCR4 in HER-2 negative breast cancer specimens predict recurrence J Surg Res 2007 141 53 9 10.1016/j.jss.2007.03.015 17574038

[B46] Andre F Cabioglu N Assi H Sabourin JC Delaloge S Sahin A Broglio K Spano JP Combadiere C Bucana C Soria JC Cristofanilli M Expression of chemokine receptors predicts the site of metastatic relapse in patients with axillary node positive primary breast cancer Ann Oncol 2006 17 945 51 10.1093/annonc/mdl053 16627550

[B47] Cabioglu N Sahin A Doucet M Yavuz E Igci A O Yildirim E Aktas E Bilgic S Kiran B Deniz G Price JE Chemokine receptor CXCR4 expression in breast cancer as a potential predictive marker of isolated tumor cells in bone marrow Clin Exp Metastasis 2005 22 39 46 10.1007/s10585-005-3222-y 16132577

[B48] Coffey JC Wang JH Smith MJF Bouchier-Hayes D Cotter TG Redmond HP Excisional surgery for cancer cure: Therapy at a cost Lancet Oncol 2003 4 760 768 10.1016/S1470-2045(03)01282-8 14662433

[B49] Galán M Viñolas N Colomer D Soler G Muñoz M Longarón R Ventura PJ Gascón P Estapé J Detection of occult breast cancer cells by amplification of CK19 mRNA by reverse transcriptase-polymerase chain reaction: role of surgical manipulation Anticancer Res 2002 22 2877 84 12530011

[B50] Topal B Aerts JL Roskams T Fieuws S Van Pelt J Vandekerckhove P Penninckx F Cancer cell dissemination during curative surgery for colorectal liver metastases Eur J Surg Oncol 2005 31 506 11 10.1016/j.ejso.2005.01.007 15922887

[B51] DiPersio JF Uy GL Yasothan U Kirkpatrick Plerixafor Nat Rev Drug Discov 2009 8 105 6 10.1038/nrd2819 19180104

